# Estimation of Primary Prevention of Gout in Men Through Modification of Obesity and Other Key Lifestyle Factors

**DOI:** 10.1001/jamanetworkopen.2020.27421

**Published:** 2020-11-24

**Authors:** Natalie McCormick, Sharan K. Rai, Na Lu, Chio Yokose, Gary C. Curhan, Hyon K. Choi

**Affiliations:** 1 Clinical Epidemiology Program, Division of Rheumatology, Allergy, and Immunology, Massachusetts General Hospital, Boston; 2 The Mongan Institute, Department of Medicine, Massachusetts General Hospital, Boston; 3 Department of Medicine, Harvard Medical School, Boston, Massachusetts; 4 Arthritis Research Canada, Richmond, British Columbia, Canada; 5 Department of Nutrition, Harvard T.H. Chan School of Public Health, Boston, Massachusetts; 6 Channing Division of Network Medicine, Department of Medicine, Brigham and Women’s Hospital, Harvard Medical School, Boston, Massachusetts; 7 Division of Renal (Kidney) Medicine, Department of Medicine, Brigham and Women’s Hospital, Harvard Medical School, Boston, Massachusetts

## Abstract

**Importance:**

The population impact of modifying obesity and other key risk factors for hyperuricemia has been estimated in cross-sectional studies; however, the proportion of incident gout cases (a clinical end point) that could be prevented by modifying such factors has not been evaluated.

**Objective:**

To estimate the proportion of incident gout cases that could be avoided through simultaneous modification of obesity and other key risk factors.

**Design, Setting, and Participants:**

The Health Professionals Follow-up Study is a US prospective cohort study of 51 529 male health professionals enrolled in 1986 and followed up through questionnaires every 2 years through 2012. Self-reported gout cases were confirmed through June 2015. Clean and complete data used for this analysis were available in June 2016, with statistical analyses performed from July 2016 to July 2019.

**Exposures:**

From data collected in the validated questionnaires, men were categorized to low-risk groups according to combinations of the following 4 factors: normal body mass index (BMI [calculated as weight in kilograms divided by height in meters squared]; <25), no alcohol intake, adherence to Dietary Approaches to Stop Hypertension (DASH)–style diet (highest quintile of DASH diet score), and no diuretic use.

**Main Outcomes and Measures:**

Population attributable risks (PARs) for incident gout meeting the preliminary American College of Rheumatology survey criteria, overall and stratified by BMI.

**Results:**

We analyzed 44 654 men (mean [SD] age, 54.0 [9.8] years) with no history of gout at baseline. During 26 years of follow-up, 1741 (3.9%) developed incident gout. Among all participants, PAR for the 4 risk factors combined (BMI, diet, alcohol use, and diuretic use) was 77% (95% CI, 56%-88%). Among men with normal weight (BMI <25.0) and overweight (BMI 25.0-29.9), we estimated that more than half of incident gout cases (69% [95% CI, 42%-83%] and 59% [95% CI, 30%-75%], respectively) may have been prevented by the combination of DASH-style diet, no alcohol intake, and no diuretic use. However, among men with obesity (BMI ≥30), PAR was substantially lower and not significant (5% [95% CI, 0%-47%]).

**Conclusions and Relevance:**

The findings of this cohort study suggest that addressing excess adiposity and other key modifiable factors has the potential to prevent the majority of incident gout cases among men. Men with obesity may not benefit from other modifications unless weight loss is addressed.

## Key Points

**Question:**

What is the estimated proportion of incident gout cases that could be prevented through modification of obesity and other key risk factors?

**Findings:**

In this cohort study of 44 654 US men followed up over 26 years, we estimated that 77% of confirmed incident gout cases may have been prevented had all men been of normal weight and adherent to a diet similar to the Dietary Approaches to Stop Hypertension pattern, with no intake of alcohol or diuretics. However, among men with obesity, modification of the other factors would not prevent gout.

**Meaning:**

These findings suggest that addressing excess adiposity and other key modifiable factors has the potential to prevent majority of incident gout cases among men.

## Teaser

This cohort study estimates the proportion of incident gout cases among US men that could be prevented through modification of obesity and other lifestyle risk factors.

## Introduction

Gout is the most common inflammatory arthritis in most Western countries,^1,2,3,4^ and gout flares are among the most painful events experienced by people.^5^ Paralleling the modern obesity epidemic^6^ (a trait of the metabolic syndrome), the disease burden of gout has been increasing worldwide,^7,8,9^ even reaching the level of a “modern gout epidemic”^10^ in Western countries, with hospitalization rates and costs due to gout doubling alongside, in the US^11^ and beyond.^12,13^

These data collectively underscore the need for primary prevention of this painful condition, which can be achieved by modifying its risk factors at the population level. A recent cross-sectional study reported a minimal variance of serum urate (SU) levels (causal precursor for gout) in the US explained by an isocaloric dietary pattern,^14^ whereas a subsequent cross-sectional analysis found a substantial proportion of hyperuricemia cases could potentially be prevented by modifying individual risk factors such as obesity, healthy dietary pattern, alcohol intake, and diuretic use.^15^ However, to date, the proportion of actual gout itself that could potentially be prevented by modifying such factors remains unknown. To address these gaps, we investigated obesity^16,17,18^ and other key risk factors simultaneously in association with the risk of incident gout and estimated the proportion of such cases that could theoretically be avoided if individuals modified sets of these risk factors.

## Methods

### Study Population and Design

The Health Professionals Follow-up Study (HPFS) is an ongoing prospective cohort study established in 1986 when 51 529 male dentists, optometrists, osteopaths, pharmacists, podiatrists, and veterinarians returned a mailed questionnaire assessing their medical history, current diet, and lifestyle habits.^19^ The men are predominantly white (91%) and were aged 40 to 75 in 1986. Questionnaires are administered every 2 years with a follow-up rate exceeding 90%. For the current analysis, we excluded men with a diagnosis of gout at baseline and used questionnaires through 2012. Self-reported gout cases were confirmed through June 2015; clean and complete data were available in June 2016 and analyzed from July 2016 to July 2019. This study was approved by the Partners Health Care System institutional review board. Completion of the self-administered questionnaire was considered to imply informed consent. This study followed the Strengthening the Reporting of Observational Studies in Epidemiology (STROBE) reporting guideline.

### Ascertainment of Lifestyle Factors

Body mass index (BMI; calculated as weight in kilograms divided by height in meters squared) was calculated using the most recently updated weight; self-reported weight has been found to be reliable (*r* = 0.97) among a subset of men who underwent direct measurement of their weight in this cohort.^20^ Beginning in 1986 and every 4 years thereafter, dietary intake was assessed using a validated food frequency questionnaire (FFQ) that inquired about the average intake of individual foods and beverages (including alcoholic beverages) consumed over the previous year. Its reproducibility and validity has been well documented in this cohort.^21,22^

### Assessment of Medications and Medical Conditions

Participants provided information on regular use of medications (including thiazide and loop diuretics) and medical conditions. As with BMI, self-reporting of these data has been found to be reliable in validation studies, and prior work has shown that these data collected biennially are able to estimate the risk of developing several diseases in this cohort, including gout.^23,24,25^

### Definition of Low-Risk Groups

As detailed in the eMethods in the Supplement, we focused on 4 common modifiable factors accepted as associated with the risk of gout (ie, obesity,^15,26,27^ alcohol,^28^ diet,^29^ and diuretic use^26,30,31^). The criteria used to define a low-risk group according to levels of each risk factor were similar to those used in previous analyses of end points related to gout, such as myocardial infarction, type 2 diabetes, and hypertension.^16,17,18^ The low-risk group for adiposity was primarily defined as BMI less than 25, as has been done previously.^16,17,18^ We additionally performed sensitivity analyses defining the low-risk group as BMI less than 23 and 27. The low-risk group for alcohol was defined as no use in our primary analysis, and consumption up to 10 g per day in a sensitivity analysis.

The Dietary Approaches to Stop Hypertension (DASH) diet is an established dietary pattern shown in multiple randomized trials to substantially reduce blood pressure.^32^ The DASH diet discourages purine-rich red meat as well as fructose-rich foods, while promoting consumption of low-fat dairy products, healthy protein sources, and vegetables/fruits,^29,33^ all of which are individually associated with a lower risk of developing incident gout.^23,25,29,34^ Calculation of the DASH-style diet score from the FFQ has been described in detail elsewhere.^29^ We considered participants to be at low risk for incident gout if they had a DASH-style diet score in the highest quintile of the cohort, as done previously for hypertension.^16^ We defined the low-risk group for diuretics as no use.

### Ascertainment of Incident Gout

On each biennial questionnaire, participants indicated whether they had received a diagnosis of gout from a physician. We mailed a supplementary questionnaire to those reporting new cases of gout diagnosed from 1986 onward to confirm the report, according to the preliminary American College of Rheumatology survey criteria for gout,^35^ and ascertain the date of gout onset.

### Statistical Analysis

We calculated person-years of follow-up for each participant using the interval between the date of the return of the 1986 questionnaire to the date of incident gout diagnosis, death, or end of the study period, whichever came first. We analyzed the association between categories of each of the 4 modifiable risk factors and the risk of incident gout using Cox proportional hazard regression models to obtain relative risks (RRs), adjusting in a time-varying manner for the other 3 factors, as well as age, total energy intake, coffee intake, vitamin C supplementation, history of kidney failure, and history of hypertension, all of which were significant risk factors for gout in previous studies, including those from the HPFS.^23,24,25,36^ If data were missing on a given questionnaire, we carried forward the value from the previous questionnaire.^37,38^ We additionally accounted for the competing risk of death, according to the method of Fine and Gray,^39^ in a sensitivity analysis.

For sufficient power for joint effect estimation and ease of interpretation, each factor was subsequently dichotomized into either low-risk or non–low-risk categories; specifically, BMI (<25 vs ≥25),^16^ alcohol intake (no intake vs any), DASH diet score (highest quintile vs others),^16^ and diuretic use (no vs yes). Given the prominent role of adiposity in gout risk,^15,26,27^ we analyzed the joint associations between BMI less than 25 and other low-risk factors with incident gout using Cox proportional hazard models. First, men with BMI less than 25 and one other low-risk lifestyle factor (no alcohol intake or DASH diet score in the highest quintile) were compared with all other men, adjusted for the above covariates and diuretic use. Then, men with a combination of BMI less than 25, no alcohol consumption, and DASH-style diet were analyzed, followed by men with these low-risk factors plus no diuretic use.

For each combination, we calculated the population attributable risk (PAR),^40^ an estimate of the percentage of incident gout cases in this population of male health professionals that could theoretically have been avoided if all men had been in the low-risk group, assuming an association between each risk factor and the outcome of developing gout.^17,18^ The PARs for individual risk factors accounted for covariates using regression models.^41^ We additionally conducted a stratified analysis according to BMI (<25.0, 25.0-29.9, and ≥30.0), comparing men in the low-risk category with all other men in each stratum.

## Results

We included 44 654 men in the final analysis (mean [SD] age at baseline of 54.0 [9.8] years), identifying 1741 incident gout cases over 26 years of follow-up (3.9% of men). The most important risk factor was BMI, with RRs of 1.29 (95% CI, 1.06-1.57), 1.90 (95% CI, 1.59-2.25), and 2.65 (95% CI, 2.18-3.22), respectively, for men with a BMI of 23.0 to 24.9 (higher end of normal), 25.0 to 29.9 (overweight), and 30.0 or greater (obesity) compared with those of BMI less than 23.0 (Table 1). In this population, 31% (95% CI, 26%-35%) of incident gout cases could be attributed to overweight or obesity (ie, BMI ≥25) alone.

**Table 1.** Individual Modifiable Risk Factors and the Relative Risk of Gout

Risk factor	Cases, No. (%)^a^	Person-years, %	Relative risk (95% CI)^b^
BMI			
<23.0	152 (8.7)	17.7	1 [Reference]
23.0-24.9	314 (18.0)	25.8	1.29 (1.06-1.57)
25.0-29.9	926 (53.2)	45.6	1.90 (1.59-2.25)
≥30.0	345 (19.8)	10.4	2.65 (2.18-3.22)
Alcohol consumption, g/d			
0	332 (19.1)	25.0	1 [Reference]
0.1-4.9	323 (18.6)	23.2	1.05 (0.90-1.23)
5.0-9.9	219 (12.6)	14.0	1.20 (1.01-1.43)
10.0-29.9	553 (31.8)	27.2	1.57 (1.37-1.81)
≥30.0	314 (18.0)	10.5	2.10 (1.79-2.46)
Quintile of DASH Diet Score			
1	397 (22.8)	19.9	1 [Reference]
2	395 (22.7)	20.2	0.94 (0.81-1.08)
3	369 (21.2)	20.2	0.91 (0.79-1.05)
4	332 (19.1)	19.8	0.86 (0.74-1.00)
5	248 (14.2)	19.9	0.74 (0.63-0.88)
Diuretic use			
No	1378 (79.1)	92.6	1 [Reference]
Yes	363 (20.9)	7.4	2.10 (1.85-2.39)

Abbreviations: BMI, body mass index (calculated as weight in kilograms divided by height in meters squared); DASH, Dietary Approaches to Stop Hypertension.

^a^ The total number of cases of gout was 1741, but because of missing values for BMI at baseline (n = 4 [0.2%]), the numbers for BMI do not add up to 1741. No other risk factors were missing at baseline.

^b^ Mutually adjusted for the other risk factors in the Table as well as age, total energy intake, coffee intake, vitamin C supplementation, history of kidney failure, and history of hypertension.

The remaining 3 factors (ie, alcohol intake, a DASH-style diet, and diuretic use) were also all individually associated with an increased risk of developing gout (Table 1). For example, 22% (95% CI, 11%-32%) of incident gout cases in this population could theoretically be prevented through adherence to a DASH-style diet (ie, having a DASH-style diet score in the top quintile). RRs were nearly identical when accounting for the competing risk of death (eTable 1 in the Supplement).

The RRs and PARs among men in the combined 2, 3, and 4 low-risk factor groups are shown in Table 2. Men in the 2 low-risk factor category (BMI <25.0 and no alcohol intake) had an RR for incident gout of 0.54 (95% CI, 0.43-0.67) as compared with all other men in the cohort. The PAR associated with this combination was 43% (95% CI, 32%-54%), suggesting that 43% of incident gout cases may have been prevented had all men been of normal weight with no alcohol intake. The PAR increased to 69% (95% CI, 47%-82%) when adherence to a DASH-style diet was added as a factor to the low-risk group, and further increased to 77% (95% CI, 56%-88%) when no diuretic use was included. After lowering the threshold for normal BMI from less than 25 to less than 23.0, the PAR increased slightly to 79% (95% CI, 44%-92%); conversely, raising the normal BMI threshold to less than 27.0 was associated with a decrease in PAR to 68% (95% CI, 50%-80%). Finally, raising the allowable alcohol intake from none to a maximum of 10 g per day was associated with a decrease in the 4 low–risk factor category PAR from 77% to 70% (95% CI, 57%-79%).

**Table 2.** Relative and Population Attributable Risks of Gout for Groups Defined by Combinations of Modifiable Risk Factors

Risk Factor^a^	Men, No. (%)	Gout cases, No.	Relative Risk (95% CI)^b^	PAR (95% CI), %
2 Factors in low-risk category				
BMI <25, no alcohol intake^c^	10 109 (22.1)	89	0.54 (0.43-0.67)	43 (32-54)
BMI<25, highest quintile of DASH diet score^d^	8192 (17.9)	94	0.65 (0.51-0.83)	33 (15-47)
3 Factors in low-risk category (BMI <25, no alcohol intake, highest quintile of DASH diet score)^e^	3072 (6.7)	13	0.31 (0.18-0.53)	69 (47-82)
4 Factors in low-risk category (BMIb<25, highest quintile of DASH diet score, no alcohol intake, no diuretic use)	2970 (6.5)	9	0.23 (0.12-0.44)	77 (56-88)

Abbreviations: BMI, body mass index (calculated as weight in kilograms divided by height in meters squared); DASH, Dietary Approaches to Stop Hypertension; PAR, population attributable risk.

^a^ Men with a missing value were considered to be in the high-risk category for that factor.

^b^ All relative risks were adjusted for age, total energy intake, coffee intake, vitamin C supplementation, history of kidney failure, and history of hypertension.

^c^ Additionally adjusted for DASH diet score and diuretic use.

^d^ Additionally adjusted for alcohol use and diuretic use.

^e^ Additionally adjusted for diuretic use.

Among those in both the normal weight and overweight categories, we estimated that more than half of cases of incident gout (69% and 59%, respectively) could theoretically have been prevented by the combination of a DASH-style diet, no alcohol intake, and no diuretic use (Table 3). However, among men with obesity, the PAR was substantially lower (5%) and not statistically significant (Table 3), suggesting that modification of these risk factors alone would not prevent the development of gout in this BMI subgroup.

**Table 3.** Relative and Population Attributable Risks of Gout Stratified by BMI

Risk Factor	Men, No. (%)	Gout cases, No.	Relative Risk (95% CI)^a^	PAR (95% CI), %
BMI <25.0				
2 Factors in low-risk category (no alcohol intake, highest quintile of DASH diet score)^b^	3072 (10.8)	13	0.39 (0.23-0.69)	59 (30-75)
3 Factors in low-risk category (no alcohol intake, highest quintile of DASH diet score, no diuretic use)	2970 (10.4)	9	0.29 (0.15-0.57)	69 (42-83)
BMI 25.0-29.9				
2 Factors in low-risk category (no alcohol intake, highest quintile of DASH diet score)^b^	2286 (7.5)	19	0.46 (0.29-0.73)	53 (27-70)
3 Factors in low-risk category (no alcohol intake, highest quintile of DASH diet score, no diuretic use)	2168 (7.2)	15	0.40 (0.24-0.67)	60 (33-75)
BMI≥30.0				
2 Factors in low-risk category (no alcohol intake, highest quintile of DASH diet score)^b^	559 (6.6)	12	0.82 (0.46-1.46)	18 (0-53)
3 Factors in low-risk category (no alcohol intake, highest quintile of DASH diet score, no diuretic use)	519 (6.1)	11	0.95 (0.51-1.74)	5 (0-47)

Abbreviations: BMI, body mass index (calculated as weight in kilograms divided by height in meters squared); DASH, Dietary Approaches to Stop Hypertension; PAR, population attributable risk.

^a^ All relative risks were adjusted for age, total energy intake, coffee intake, vitamin C supplementation, history of kidney failure, and history of hypertension.

^b^ Additionally adjusted for diuretic use.

## Discussion

In this large prospective cohort study of men, the combination of low-risk modifiable factors, including maintenance of normal BMI, avoiding alcohol consumption, eating a diet high in fruits, vegetables, and low-fat dairy products as well as low in red/processed meats and sweetened beverages, and no diuretic use, was associated with a substantially lower risk of incident gout during follow-up. We estimated that 77% of incident cases in this population hypothetically could have been avoided by modifying these key risk factors. Our longitudinal analysis overcomes the potential limitations of cross-sectional designs and intermediate surrogate end points^14,15^ and supports the prominent role of these modifiable lifestyle factors in explaining the increasing incidence and prevalence of gout in multiple settings worldwide.^7^

Our findings might appear at odds with a recent report of minimal variance in serum urate explained by isocaloric diets in the US population.^14^ However, that study was limited to only 1 risk factor (ie, isocaloric dietary pattern) and used a cross-sectional design with the outcome of serum urate, whereas the current study examined obesity and the 3 other key factors in combination, and was a prospective longitudinal study with a clinical end point of incident gout cases. As such, modifiable lifestyle factors may affect the risk of gout beyond their influence on serum urate levels, such as affecting epigenetics or adenosine monophosphate–activated protein kinase activities.^42^ Moreover, many statistical methods papers, including the latest for environmental exposures^15^ and genetics,^43^ have shown limitations of so-called variance explained (or heritability measure in genetics^43^) as an effect measure for highly prevalent exposures such as obesity and Western diets in the US^15^ or extremely common genes^43^ (where there is near-zero variability in the exposures, owing to their ubiquity^44^).

Excess adiposity was the most important risk factor for developing gout and the largest individual contributor to the PAR. A potential causal relationship between obesity, serum urate level, and the risk of gout is supported by its mechanistic plausibility,^45,46,47,48,49,50^ prospective cohort studies,^26,27,51^ Mendelian randomization studies,^52,53^ and weight loss interventions through bariatric surgery^54,55^ or lifestyle modification.^54^ As such, global obesity epidemic trends^6^ have coincided with the rising gout burden^7^; there is compelling evidence to suggest that the worsening obesity epidemic is largely driven by dietary and lifestyle changes occurring over the past several decades.^56^ In particular, as diet (calorie source) and physical activity (modifiable calorie output) play essential roles in the risk of obesity, the substantial PAR associated with BMI alone suggests a prominent role of diet and exercise, through modifying BMI, on the risk of gout at the population level.

Given the importance of adiposity in gout risk, we also tested whether participants with overweight or obesity who otherwise adhered to combinations of the remaining modifiable factors had a reduced risk of incident gout. The combination of consuming no alcohol, having a DASH score in the highest quintile, and using no diuretics may have avoided 59% of incident gout cases among men who were overweight, whereas the same combination may have avoided 69% of cases among men with BMI less than 25.0. However, we found no significant contribution from these modifiable factors among individuals with obesity, suggesting that for the risk of gout, men with obesity may not benefit from the other modifications unless weight loss is also addressed. This finding is particularly relevant because obesity is common (eg, approximately one-third of the US population) and in the absence of calorie restriction and physical activity, adherence to the other 3 risk factors may not address excess adiposity.

Beyond the role of diet as the calorie source determinant, the isocaloric role of individual food items and dietary patterns (ie, food quality rather than quantity) in developing gout has also been prospectively documented.^23,24,25,29,36,57,58^ In our analysis, as opposed to individual food items, we focused on the potential population impact of an entire eating pattern (ie, the DASH diet pattern), which corresponds well to the individual food or nutrient items with antigout properties,^23,25,29,34^ and better reflects the way foods are consumed together in reality. Our findings suggest that 22% of incident gout cases could theoretically be prevented by adherence to a DASH-style eating pattern alone (ie, a DASH-style diet score in the top quintile), supporting a considerable role of this isocaloric dietary pattern in reducing incident gout risk. Moreover, adoption of the DASH diet could further prevent and treat related conditions including hypertension, which affects nearly 75% of patients with gout.^59^

The potential benefits of modifying lifestyle factors documented in this study are consistent with the PAR findings for comorbidities of gout.^16,17,18,60^ For example, prior work has found that adherence to healthy diet and lifestyle guidelines could have prevented 91% of incident cases of type 2 diabetes^18^ and 78% of incident hypertension^16^ among women. Taken together, our findings embolden the shared role of lifestyle factors for developing these cardiovascular-metabolic conditions and further strengthen public health recommendations to maintain a normal BMI and follow healthy lifestyle guidelines to simultaneously help prevent the majority of cases of these serious and costly conditions altogether.^16,17,18,60^ Although beyond the scope of this article, the interplay between these lifestyle factors and genetic polymorphisms associated with gout risk should be examined in future studies.

Diuretics increase the net reabsorption of uric acid in the proximal tubule of the nephron and thereby reduce the urinary excretion and increase the risk of hyperuricemia^61,62^ and gout.^26,30^ To that end, urate-lowering antihypertensive agents (eg, calcium channel blockers or losartan) could be preferred to minimize gout risk. As our findings are based on the reference point of no diuretic use, the gout risk difference compared with urate-lowering antihypertensive agents could be even larger.

A key strength of the current study was the large number of confirmed incident gout cases. The validity of gout ascertainment in these cohorts of health professionals has been documented by the high degree of concordance with medical record review.^23^ We obtained high-quality data with minimal loss to follow-up over the study period. Furthermore, dietary and lifestyle data were prospectively collected before the diagnosis of gout, minimizing the potential for biased recall of these factors.

### Limitations

While we adjusted for potential confounders, as in any observational study, our estimates remain subject to residual and unmeasured confounding. The exact dates of gout diagnosis and change in values of time-varying covariates between questionnaires were not available, but this interval censoring problem, a type of nondifferential measurement error, would bias our RR estimates toward the null, leading to more conservative PAR estimates. Our findings can be extrapolated most directly to white men over the age of 40 years with no history of gout, and while men are at substantially greater risk for developing gout than women, these data generated from male health professionals may not apply to the general population. However, as the risk factors for gout tend to be more common in the general population,^59^ the magnitude of the risk reduction would probably be even greater than that observed in this study. For example, the PAR of maintaining a normal BMI would be 35% using the prevalence of obesity from the NHANES (2006-2017), as compared with 31% in our cohort. Moreover, while we could not estimate the joint effects of these risk factors on serum urate levels (not measured in the HPFS), our findings are consistent with our recent analysis of modifiable risk factors for hyperuricemia (the causal precursor to gout) among a nationally-representative sample of US men and women.^15^ There, 44%, 9%, and 8% of hyperuricemia cases could be attributed to overweight/obesity, unhealthy diet, and alcohol, respectively.

## Conclusions

Our findings from this cohort of men suggest the majority of gout cases could potentially be prevented by achieving and maintaining a normal weight, avoiding alcohol consumption, eating a DASH-style diet, and avoiding diuretic use. Men with obesity may not benefit from other modifications unless weight loss is addressed.

## Notes

Supplement. **eTable 1.** Individual Modifiable Risk Factors and the Relative Risk of Gout, Accounting for the Competing Risk of Death **eMethods.** Detailed Methods **eReferences.**
