# Supplementary material for: Estimation of Primary Prevention of Gout in Men Through Modification of Obesity and Other Key Lifestyle Factors
Source: JAMA Netw Open. 2020 Nov 24;3(11):e2027421. doi: 10.1001/jamanetworkopen.2020.27421 (PMC7686865; doi:10.1001/jamanetworkopen.2020.27421)
Supplement: Supplement. — eTable 1. Individual Modifiable Risk Factors and the Relative Risk of Gout, Accounting for the Competing Risk of Death eMethods. Detailed Methods eReferences. [file jamanetwopen-e2027421-s001.pdf]

Supplemental Online Content

McCormick N, Rai SK, Lu N, Yokose C, Curhan GC, Choi HK. Estimation of primary prevention of gout in men through modification of obesity and other key lifestyle factors. *JAMA Netw Open*. 2020;3(11):e2027421. doi:10.1001/jamanetworkopen.2020.27421

eTable 1. Individual Modifiable Risk Factors and the Relative Risk of Gout, Accounting for the Competing Risk of Death

eMethods. Detailed Methods

eReferences.

This supplemental material has been provided by the authors to give readers additional information about their work.

© 2020 McCormick N et al. *JAMA Network Open.*

eTable 1. Individual Modifiable Risk Factors and the Relative Risk of Gout, Accounting for the Competing Risk of Death

Risk Factor	Number of Cases (%)*	Percentage of Person-Years	Relative Risk (95% CI)**
**Body Mass Index (kg/m^2)**			
<23.0	152 (8.7)	17.7	1.0
23.0-24.9	314 (18.0)	25.8	1.28 (1.06, 1.56)
25.0-29.9	926 (53.2)	45.6	1.89 (1.59, 2.24)
≥30.0	345 (19.8)	10.4	2.63 (2.16, 3.19)
**Alcohol Consumption (g/day)**			
0	332 (19.1)	25.0	1.0
0.1-4.9	323 (18.6)	23.2	1.04 (0.89, 1.22)
5.0-9.9	219 (12.6)	14.0	1.20 (1.01, 1.42)
10.0-29.9	553 (31.8)	27.2	1.57 (1.36, 1.80)
≥30.0	314 (18.0)	10.5	2.09 (1.78, 2.45)
**Quintile of DASH Diet Score**			
1st	397 (22.8)	19.9	1.0
2nd	395 (22.7)	20.2	0.93 (0.81, 1.07)
3rd	369 (21.2)	20.2	0.91 (0.79, 1.05)
4th	332 (19.1)	19.8	0.86 (0.74, 1.00)
5th	248 (14.2)	19.9	0.74 (0.63, 0.87)
**Diuretic Use**			
No	1378 (79.1)	92.6	1.0
Yes	363 (20.9)	7.4	2.12 (1.86 to 2.41)
Abbreviations: CI, confidence interval. DASH, Dietary Approaches to Stop Hypertension.			

© 2020 McCormick N et al. *JAMA Network Open.*

*The total number of cases of gout was 1741, but because of missing values (n=4, 0.2%) the numbers for BMI do not add up to 1741.

**Mutually adjusted for the other risk factors in the table as well as age, total energy intake, coffee intake, vitamin C supplementation, history of renal failure, and history of hypertension.

© 2020 McCormick N et al. *JAMA Network Open.*

eMethods. Detailed Methods

Risk Factors and Definition of Low-Risk Groups

While many factors have been associated with the risk of gout,[1-7] we focused on four common modifiable factors that are accepted as causally associated with the risk of gout (i.e., obesity[4,8,9], alcohol[10], diet[3], and diuretic use[4]). The criteria used to define a low-risk group according to levels of each risk factor were similar to those used in previous analyses of endpoints related to gout, such as myocardial infarction, type 2 diabetes, and hypertension.[11-13]

Assessment of Adiposity

Every two years, body mass index (BMI) was assessed. BMI was calculated using the most recently updated weight in kilograms divided by height in meters squared; self-reported weight has been found to be reliable (r=0.97) among a subset of regionally residing men who underwent direct measurement of their weight in this cohort.[14]

The low-risk group for adiposity was defined as those who had a BMI less than 25, as has been done previously,[11-13] which is defined by the World Health Organisation as the cut-point separating those of normal weight from those who are considered overweight.[17] Excess adiposity increases gout risk, both through decreased renal urate excretion and increased urate production.[18-23] Mendelian randomization studies have found obesity to be causally associated with serum urate levels,[24,25] and weight loss through bariatric surgery or lifestyle intervention leads to reductions in serum urate.[26] We additionally performed sensitivity analyses defining the low-risk group as those with a BMI less than 23 and 27.

Assessment of Diet and Alcohol

Beginning in 1986 and every four years thereafter, dietary intake was assessed using a validated food frequency questionnaire (FFQ) that inquired about the average intake of individual foods

© 2020 McCormick N et al. *JAMA Network Open.*

and beverages (including alcoholic beverages) consumed over the previous year. The current version of the questionnaire administered to participants includes more than 130 individual foods, as well as supplemental vitamins, minerals, and an open-ended section for foods not specified on the questionnaire. The reproducibility and validity of the FFQ has been well documented in this cohort.[27,28]

The low-risk group for alcohol was defined as no use in our primary analysis. In addition to physiologic loading experiments of alcohol documenting serum urate-raising effects,[29] population studies have confirmed that alcohol intake is associated with higher serum urate levels[30,31] and an increased risk of incident[10] as well as recurrent gout.[32] We additionally performed a sensitivity analysis re-defining the low-risk group as including those who consumed up to 10g of alcohol per day.

Data collected in the FFQ was used to calculate a Dietary Approaches to Hypertension (DASH)-style diet score, as described in detail elsewhere.[3] The DASH diet is an established dietary pattern that has been shown in multiple randomised trials to substantially reduce blood pressure.[33] It discourages purine-rich red meat as well as fructose-rich foods, while promoting the consumption of low-fat dairy products, healthy protein sources (including nuts and legumes), and vegetables/fruits,[3,34] all of which are individually associated with a lower risk of developing incident gout.[1-3,35] Metabolic loading experiments of purine[36-39] and fructose[40-43] have confirmed their serum urate-raising effect,[36-43] whereas dairy products showed urate-lowering effects in three experimental studies, including two randomised trials.[44-46] A higher DASH-style diet score is associated with a lower risk of incident gout, whereas the Western dietary pattern is associated with a higher risk.[3] Furthermore, a DASH diet trial analysis found that the DASH diet lowers serum urate levels compared with a typical American diet (i.e., control diet), particularly among

© 2020 McCormick N et al. *JAMA Network Open.*

those with hyperuricemia (i.e., by 1.0 mg/dL in those with a baseline serum urate level ≥6 mg/dL, and by 1.3 mg/dL in those with a baseline serum urate level ≥7 mg/dL).[47] For diet, we considered participants to be at a low risk for incident gout if they had a DASH-style diet score in the highest quintile of the cohort, as done previously for hypertension.[11]

Assessment of Medications and Medical Conditions

Participants provided information on the regular use of medications (including thiazide and loop diuretics) and medical conditions. As with BMI, self-reporting of these data have also been found to be reliable in validation studies, and prior work has shown that these data collected biennially are able to predict the risk of developing several diseases in this cohort, including gout.[1,2,7]

We defined the low-risk group for diuretics as no use. The increase in serum urate level caused by diuretics has been documented within a few days after the initiation of diuretics,[48-50] and diuretic use has been associated with an increased risk of incident gout.[4,50,51]

Ascertainment of Incident Gout

We ascertained incident cases of gout according to the preliminary American College of Rheumatology (ACR) survey criteria for gout.[52] On each biennial questionnaire, participants indicated whether they had received a diagnosis of gout from a physician. We mailed a supplementary questionnaire to those reporting new cases of gout diagnosed from 1986 onward to confirm the report and ascertain the preliminary ACR gout survey criteria and the date of gout onset.[52]

The primary endpoint of our study was incident gout fulfilling at least 6 of the 11 ACR survey criteria for gout (i.e., more than one attack of acute arthritis, maximal inflammation

© 2020 McCormick N et al. *JAMA Network Open.*

developing within one day, attack of oligoarthritis, redness observed over joints, painful or swollen first metatarsophalangeal joint, unilateral attack in first metatarsophalangeal joint, unilateral attack in a tarsal joint, tophus, hyperuricemia, asymmetric swelling within a joint, and complete termination of an attack).[52] As described in detail previously for this cohort, two board-certified rheumatologists conducted a medical record review of a sample of 50 of the men who reported a gout diagnosis, and the concordance rate between the ACR survey criteria and the medical record review was 94%.[1]

Statistical Analysis

We calculated person-years of follow-up for each participant using the interval between the date of the return of the 1986 questionnaire to the date of incident gout diagnosis, death, or the end of the study period, whichever came first. We analysed the association between each of the four modifiable risk factors and the risk of incident gout. We used Cox proportional hazard regression models to obtain relative risks (RRs) for categories of each factor, adjusting in a time-varying manner for the other three factors, as well as age, total energy intake, coffee intake, vitamin C supplementation, history of renal failure, and history of hypertension.

Each of these factors was subsequently dichotomized into either low-risk or non-low-risk categories; specifically, BMI (<25 kg/m^2 vs. ≥ 25 kg/m^2),[11] alcohol intake (no intake vs. any intake), DASH diet score (highest quintile vs. the lower four quintiles),[11] and diuretic use (no vs. yes). We analysed the joint associations between BMI <25 kg/m^2 and other low-risk factors with incident gout using Cox proportional hazard models. First, men with BMI <25 kg/m^2 and one other low-risk lifestyle factor (no alcohol intake or DASH diet score in the highest quintile) were compared with all other men, adjusting for age, total energy intake, coffee intake, vitamin C supplementation, histories of renal failure and hypertension, as well as DASH diet score (or

© 2020 McCormick N et al. *JAMA Network Open.*

alcohol use) and diuretic use. Then, men with a combination of BMI <25 kg/m^2, no alcohol consumption, and DASH-style diet were analysed. Finally, men with all three of these low-risk factors plus no diuretic use were analysed.

For each combination of low-risk factors, we calculated the population attributable risk (PAR),[53] which is an estimate of the percentage of incident gout cases in this population of male health professionals that would theoretically have been avoided if all men had been in the low-risk group, assuming a causal relation between each risk factor and the outcome of developing gout[12,13]. The PAR formula that we used was:

$$\sum_{i=0}^{k} pd_i \left(\frac{RR_i - 1}{RR_i}\right) = 1 - \sum_{i=0}^{k} \frac{pd_i}{RR_i}$$

where pd_i = the proportion of cases falling into ith exposure level and RR_i = the RR comparing the ith exposure level with the unexposed group (i = 0).[54] The PARs for individual risk factors accounted for covariates using regression models.[55] Given the prominent role of adiposity in gout risk,[4,8,9] we additionally conducted a stratified analysis according to BMI (<25.0 kg/m^2, 25.0-29.9 kg/m^2, and ≥30.0 kg/m^2), comparing men in the low-risk category with all other men in each stratum.

eReferences

1. Choi HK, Atkinson K, Karlson EW, Willett W, Curhan G. Purine-rich foods, dairy and protein intake, and the risk of gout in men. *The New England journal of medicine.* 2004;350(11):1093-1103.

2. Choi HK, Curhan G. Soft drinks, fructose consumption, and the risk of gout in men: prospective cohort study. *BMJ (Clinical research ed).* 2008;336(7639):309-312.

3. Rai SK, Fung TT, Lu N, Keller SF, Curhan GC, Choi HK. The Dietary Approaches to Stop Hypertension (DASH) diet, Western diet, and risk of gout in men: prospective cohort study. *BMJ.* 2017;357:j1794.

© 2020 McCormick N et al. *JAMA Network Open.*

4. Choi HK, Atkinson K, Karlson EW, Curhan G. Obesity, weight change, hypertension, diuretic use, and risk of gout in men: The health professionals follow-up study. *Archives of Internal Medicine.* 2005;165(7):742-748.

5. Choi HK, Willett W, Curhan G. Coffee consumption and risk of incident gout in men: A prospective study. *Arthritis Rheum.* 2007;56(6):2049-2055.

6. Choi HK, Curhan G. Coffee consumption and risk of incident gout in women: the Nurses' Health Study. *Am J Clin Nutr.* 2010;92(4):922-927.

7. Choi HK, Gao X, Curhan G. Vitamin C intake and the risk of gout in men: a prospective study. *Arch Intern Med.* 2009;169(5):502-507.

8. Roubenoff R, Klag MJ, Mead LA, Liang KY, Seidler AJ, Hochberg MC. Incidence and risk factors for gout in white men. *JAMA.* 1991;266(21):3004-3007.

9. Choi HK, McCormick N, Lu N, Rai SK, Yokose C, Zhang Y. Population Impact Attributable to Modifiable Risk Factors for Hyperuricemia. *Arthritis & rheumatology.* 2019.

10. Choi HK, Atkinson K, Karlson EW, Willett WC, Curhan G. Alcohol Intake and Risk of Incident Gout in Men - A Prospective Study. *Lancet.* 2004;363:1277-1281.

11. Forman JP, Stampfer MJ, Curhan GC. Diet and lifestyle risk factors associated with incident hypertension in women. *JAMA.* 2009;302(4):401-411.

12. Stampfer MJ, Hu FB, Manson JE, Rimm EB, Willett WC. Primary prevention of coronary heart disease in women through diet and lifestyle. *New England Journal of Medicine.* 2000;343(1):16-22.

13. Hu FB, Manson JE, Stampfer MJ, et al. Diet, lifestyle, and the risk of type 2 diabetes mellitus in women. *New England Journal of Medicine.* 2001;345(11):790-797.

14. Rimm EB, Stampfer MJ, Colditz GA, Chute CG, Litin LB, Willett WC. Validity of self-reported waist and hip circumferences in men and women. *Epidemiology (Cambridge, Mass).* 1990;1(6):466-473.

15. Ferraro PM, Taylor EN, Gambaro G, Curhan GC. Dietary and Lifestyle Risk Factors Associated with Incident Kidney Stones in Men and Women. *J Urol.* 2017;198(4):858-863.

16. Aune D, Norat T, Vatten LJ. Body mass index and the risk of gout: a systematic review and dose-response meta-analysis of prospective studies. *European journal of nutrition.* 2014;53(8):1591-1601.

© 2020 McCormick N et al. *JAMA Network Open.*

17. Gibson T, Rodgers AV, Simmonds HA, Court-Brown F, Todd E, Meilton V. A controlled study of diet in patients with gout. *Annals of the Rheumatic Diseases.* 1983;42(2):123-127.

18. Fam AG. Gout, diet, and the insulin resistance syndrome. *The Journal of rheumatology.* 2002;29(7):1350-1355.

19. Emmerson BT. The management of gout. *N Engl J Med.* 1996;334(7):445-451.

20. Emmerson BT. Alteration of urate metabolism by weight reduction. *Aust N Z J Med.* 1973;3(4):410-412.

21. Modan M, Halkin H, Fuchs Z, et al. Hyperinsulinemia--a link between glucose intolerance, obesity, hypertension, dyslipoproteinemia, elevated serum uric acid and internal cation imbalance. *Diabete Metab.* 1987;13(3 Pt 2):375-380.

22. Facchini F, Chen YD, Hollenbeck CB, Reaven GM. Relationship between resistance to insulin-mediated glucose uptake, urinary uric acid clearance, and plasma uric acid concentration. *JAMA.* 1991;266(21):3008-3011.

23. Yamashita S, Matsuzawa Y, Tokunaga K, Fujioka S, Tarui S. Studies on the impaired metabolism of uric acid in obese subjects: marked reduction of renal urate excretion and its improvement by a low-calorie diet. *Int J Obes.* 1986;10(4):255-264.

24. Lyngdoh T, Vuistiner P, Marques-Vidal P, et al. Serum uric acid and adiposity: deciphering causality using a bidirectional Mendelian randomization approach. *PLoS One.* 2012;7(6):e39321.

25. Oikonen M, Wendelin-Saarenhovi M, Lyytikainen LP, et al. Associations between serum uric acid and markers of subclinical atherosclerosis in young adults. The cardiovascular risk in Young Finns study. *Atherosclerosis.* 2012;223(2):497-503.

26. Nielsen SM, Bartels EM, Henriksen M, et al. Weight loss for overweight and obese individuals with gout: a systematic review of longitudinal studies. *Annals of the rheumatic diseases.* 2017;76(11):1870-1882.

27. Rimm EB, Giovannucci EL, Stampfer MJ, Colditz GA, Litin LB, Willett WC. Reproducibility and validity of an expanded self-administered semiquantitative food frequency questionnaire among male health professionals. *American journal of epidemiology.* 1992;135(10):1114-1126; discussion 1127-1136.

28. Yuan C, Spiegelman D, Rimm EB, et al. Validity of a Dietary Questionnaire Assessed by Comparison With Multiple Weighed Dietary Records or 24-Hour Recalls. *Am J Epidemiol.* 2017;185(7):570-584.

29. Gibson T, Rodgers AV, Simmonds HA, Toseland P. Beer drinking and its effect on uric acid. *Br J Rheumatol.* 1984;23(3):203-209.

© 2020 McCormick N et al. *JAMA Network Open.*

30. Choi HK, Curhan G. Beer, Liquor, Wine, and Serum Uric Acid Level - The Third National Health and Nutrition Examination Survey. *Arthritis Rheum.* 2004;51(6):1023-1029.

31. Major TJ, Topless RK, Dalbeth N, Merriman TR. Evaluation of the diet wide contribution to serum urate levels: meta-analysis of population based cohorts. *BMJ.* 2018;363:k3951.

32. Neogi T, Chen C, Niu J, Chaisson C, Hunter DJ, Zhang Y. Alcohol quantity and type on risk of recurrent gout attacks: an internet-based case-crossover study. *Am J Med.* 2014;127(4):311-318.

33. Appel LJ, Moore TJ, Obarzanek E, et al. A clinical trial of the effects of dietary patterns on blood pressure. DASH Collaborative Research Group. *N Engl J Med.* 1997;336(16):1117-1124.

34. Fung TT, Chiuve SE, McCullough ML, Rexrode KM, Logroscino G, Hu FB. Adherence to a DASH-style diet and risk of coronary heart disease and stroke in women. *Arch Intern Med.* 2008;168(7):713-720.

35. Choi HK, Willett W, Curhan G. Fructose-rich beverages and risk of gout in women. *JAMA.* 2010;304(20):2270-2278.

36. Clifford AJ, Riumallo JA, Young VR, Scrimshaw NS. Effects of oral purines on serum and urinary uric acid of normal, hyperuricaemic and gouty humans. *J Nutr.* 1976;106:428-450.

37. Clifford AJ, Story DL. Levels of purines in foods and their metabolic effects in rats. *J Nutr.* 1976;106:435-442.

38. Zollner N. Influence of various purines on uric acid metabolism. *Bibl Nutr Dieta.* 1973(19):34-43.

39. Zollner N, Griebsch A. Diet and gout. *Adv Exp Med Biol.* 1974;41:435-442.

40. Stirpe F, Della Corte E, Bonetti E, Abbondanza A, Abbati A, De Stefano F. Fructose-induced hyperuricaemia. *Lancet.* 1970;2(7686):1310-1311.

41. Emmerson BT. Effect of oral fructose on urate production. *Ann Rheum Dis.* 1974;33(3):276-280.

42. Perheentupa J, Raivio K. Fructose-induced hyperuricaemia. *Lancet.* 1967;2(7515):528-531.

43. Dalbeth N, House ME, Gamble GD, et al. Population-specific influence of SLC2A9 genotype on the acute hyperuricaemic response to a fructose load. *Ann Rheum Dis.* 2013;72(11):1868-1873.

© 2020 McCormick N et al. *JAMA Network Open.*

44. Garrel DR, Verdy M, PetitClerc C, Martin C, Brule D, Hamet P. Milk- and soy-protein ingestion: acute effect on serum uric acid concentration. *Am J Clin Nutr.* 1991;53(3):665-669.

45. Ghadirian P, Shatenstein B, Verdy M, Hamet P. The influence of dairy products on plasma uric acid in women. *Eur J Epidemiol.* 1995;11(3):275-281.

46. Dalbeth N, Wong S, Gamble GD, et al. Acute effect of milk on serum urate concentrations: a randomised controlled crossover trial. *Ann Rheum Dis.*69(9):1677-1682.

47. Juraschek SP, Gelber AC, Choi HK, Appel LJ, Miller ER, 3rd. Effects of the Dietary Approaches To Stop Hypertension (DASH) Diet and Sodium Intake on Serum Uric Acid. *Arthritis & rheumatology.* 2016.

48. Leary WP, Reyes AJ, Wynne RD, van der Byl K. Renal excretory actions of furosemide, of hydrochlorothiazide and of the vasodilator flosequinan in healthy subjects. *J Int Med Res.* 1990;18(2):120-141.

49. Hunter DJ, York M, Chaisson CE, Woods R, Niu J, Zhang Y. Recent diuretic use and the risk of recurrent gout attacks: the online case-crossover gout study. *J Rheumatol.* 2006;33(7):1341-1345.

50. Choi HK, Soriano LC, Zhang Y, Rodriguez LA. Antihypertensive drugs and risk of incident gout among patients with hypertension: population based case-control study. *BMJ.* 2012;344:d8190.

51. Bruderer S, Bodmer M, Jick SS, Meier CR. Use of diuretics and risk of incident gout: a population-based case-control study. *Arthritis & rheumatology.* 2014;66(1):185-196.

52. Wallace SL, Robinson H, Masi AT, Decker JL, McCarty DJ, Yu TF. Preliminary criteria for the classification of the acute arthritis of primary gout. *Arthritis and rheumatism.* 1977;20(3):895-900.

53. Spiegelman D, Hertzmark E, Wand HC. Point and interval estimates of partial population attributable risks in cohort studies: examples and software. *Cancer Causes Control.* 2007;18(5):571-579.

54. Rockhill B, Newman B, Weinberg C. Use and misuse of population attributable fractions. *Am J Public Health.* 1998;88(1):15-19.

55. Benichou J. A review of adjusted estimators of attributable risk. *Stat Methods Med Res.* 2001;10(3):195-216.

© 2020 McCormick N et al. *JAMA Network Open.*
